# Data Resource Profile: EULAT Eradicate GBC: the European-Latin American Research Consortium towards Eradication of Preventable Gallbladder Cancer

**DOI:** 10.1093/ije/dyaf127

**Published:** 2025-07-29

**Authors:** Dominique Scherer, Carol Barahona Ponce, Claudio Mengoa, Paola Montenegro, Hector Losada, Ana Lineth Garcia, Armando Rojas, Erik Morales, Allan Vera Kortmann, Loreto Spencer, Alejandro Ortega, Karina Vargas Valdebenito, Juan Carlos Roa, Cristina Inklemona, Alicia Colombo Flores, Romy Kirsten, Linda Zollner, Katherine Marcelain, Trine B Rounge, Hilde Langseth, Sarah Jane Lewis, Gerardo Francisco Arroyo, Ricardo Armisen, Bruno Nervi Nattero, Bettina G Muller, Piga Roxana Fernández Kaempffer, Rajiv Kumar, Pamela Salinas-Alvarez, Rachel Sabine Kelly, Mazda Jenab, Justo Lorenzo Bermejo

**Affiliations:** Statistical Genetics Research Group, Institute of Medical Biometry, Heidelberg University, Heidelberg, Germany; Statistical Genetics Research Group, Institute of Medical Biometry, Heidelberg University, Heidelberg, Germany; Instituto Regional de Enfermedades Neoplásicas, Arequipa, Peru; Departamento de Medicina Oncológica, Instituto Nacional de Enfermedades Neoplásicas, Lima, Peru; Departamento de Cirugía, Universidad de la Frontera, Temuco, Chile; Instituto de Investigaciones Biomédicas (IIBISMED), Facultad de Medicina, Universidad Mayor de San Simón, Cochabamba, Bolivia; Facultad de Medicina, Universidad Católica del Maule, Talca, Chile; Facultad de Medicina, Universidad Católica del Maule, Talca, Chile; Servicio de Anatomia Patologica, Hospital Regional de Talca, Talca, Chile; Hospital de Puerto Montt, Los Lagos, Puerto Montt, Chile; Hospital Clinico Regional Concepción, Concepción, Chile; Hospital Juan Noé Crevani, Arica, Chile; Hospital Juan Noé Crevani, Arica, Chile; Center for Cancer Prevention and Control (CECAN), Chile; Servicio de Anatomia Patologica, Pontificia Universidad Catolica de Chile, Santiago, Chile; Hospital Pablo Soria, San Salvador de Jujuy, Argentina; Center for Cancer Prevention and Control (CECAN), Chile; Department of Anatomy Pathology, Faculty of Medicine, Universidad de Chile, Santiago, Chile; Pathological Anatomy Service, Clinical Hospital of the University of Chile, Santiago, Chile; Departamento de Oncología Básico Clínica, Facultad de Medicina, Universidad de Chile, Santiago, Chile; University Hospital Mannheim, Medicial Faculty Mannheim, Heidelberg University, Mannheim, Germany; Statistical Genetics Research Group, Institute of Medical Biometry, Heidelberg University, Heidelberg, Germany; Center for Cancer Prevention and Control (CECAN), Chile; Departamento de Oncología Básico Clínica, Facultad de Medicina, Universidad de Chile, Santiago, Chile; Centre for Bioinformatics, Department of Pharmacy, University of Oslo, Oslo, Norway; Department of Research, Cancer Registry of Norway, Oslo, Norway; Department of Research, Cancer Registry of Norway, Oslo, Norway; Department of Population Health Sciences, University of Bristol, Bristol, United Kingdom; ILOGI (Latin-American Gastrointestinal Oncology Intergroup), San Salvador de Jujuy, Argentina; Centro de Genética y Genómica, Instituto de Ciencias e Innovación en Medicina, Facultad de Medicina Clínica Alemana Universidad del Desarrollo, Santiago, Chile; Center for Cancer Prevention and Control (CECAN), Chile; Departamento de Hematología y Oncología, Pontificia Universidad Católica De Chile, Santiago, Chile; Chilean Cooperative Group for Oncological Research (GOCCHI), Santiago, Chile; Fundación GIST Chile, Santiago, Chile; Statistical Genetics Research Group, Institute of Medical Biometry, Heidelberg University, Heidelberg, Germany; Instituto de Alta Investigación, Universidad de Tarapacá, Arica, Chile; Channing Division of Network Medicine, Brigham and Women’s Hospital and Harvard Medical School, Boston, USA; Nutrition and Metabolism Branch, International Agency for Research on Cancer (IARC-WHO), Lyon, France; Statistical Genetics Research Group, Institute of Medical Biometry, Heidelberg University, Heidelberg, Germany; Laboratory of Biostatistics for Precision Oncology, Institut de cancérologie Strasbourg Europe, Strasbourg, France

**Keywords:** gallbladder cancer, personalized prevention, risk prediction, biorepository

Key Features

- The European-Latin American Research Consortium towards Eradication of Preventable Gallbladder Cancer (EULAT Eradicate GBC) has established a biorepository of >14 000 individuals as part of a Latin American gallbladder cancer (GBC) case–control study and a GBC case–control study nested within large European prospective cohorts to discover and validate biomarkers for GBC prevention.
- The EULAT Eradicate GBC is unique in its study design, in the combination of European and Latin American data and samples, and in the sampling of Latin American patients prior to cholecystectomy.
- Samples are used for molecular-genetic profiling of the study participants and complemented with clinical, lifestyle, cultural, and behavioral data with the aim of generating individual GBC risk scores.
- Between January 2020 and May 2025, >11 400 Latin American and >300 European individuals were included in the study, with an age range of 18–96 years.
- Research collaboration requests and applications for access to data and samples can be sent to EULAT_Eradicate_GBC@imbi.uni-heidelberg.de.

## Data resource basics

Gallbladder cancer (GBC, ICD-10 diagnosis code C23) is a relatively rare but aggressive disease that is expected to cause 95 609 deaths worldwide in 2025 (https://gco.iarc.fr/en). Most GBC deaths occur in low- to middle-income countries, including northern and north-eastern India, Bolivia, Chile, and the surrounding Andean regions. The research into this aggressive malignancy has remained neglected for myriad reasons [1]. Host factors that increase GBC risk include age, female sex, gallstones, Indigenous American ancestry, family history of GBC, and body mass index (BMI) [2, 3]. However, individual GBC risk prediction for primary prevention remains unreliable.

Surgery offers the potential to cure GBC confined to the mucosa; however, early symptoms of GBC are non-specific and, currently, there is a dearth of tests to detect gallbladder tumors early. Less than 20% of patients qualify for curative surgery at diagnosis, translating into an average 5-year survival rate of only 2% for patients with metastatic disease [4].

To improve the prevention of GBC, in 2019, we initiated the establishment of a European-Latin American research consortium— the European-Latin American Research Consortium towards Eradication of Preventable Gallbladder Cancer (EULAT Eradicate GBC), funded by the European Union’s Horizon2020 program and coordinated from Heidelberg University Hospital, Germany, with the ambitious goal of eradicating preventable GBC (https://cordis.europa.eu/project/id/825741, https://www.klinikum.uni-heidelberg.de/eulat-eradicate-gbc). The EULAT Eradicate GBC combines a multinational case–control study of Latin American patients with GBC and gallstone disease with a multinational case–control study nested within large European cohorts. This combined study design allows biomarker discovery in a case–control setting, followed by prospective validation in independent samples. Furthermore, it facilitates a comparative analysis of biomarkers of GBC risk in high-incidence (Latin America) and low-incidence (Europe) geographic regions.

Following standard operating procedures and taking advantage of a web-based application for the electronic collection of socio-demographic, sample, clinical, and lifestyle data, the EULAT Eradicate GBC is building a comprehensive biorepository integrated into a specifically designed IT platform with high-quality data and samples to pursue the following specific objectives:

- identifying biomarkers of GBC risk that guide prophylactic cholecystectomy and translate into novel and efficient strategies for precision prevention;
- improving the current understanding of the causal mechanisms that link lifestyle, cultural, and behavioral factors to GBC development;
- unraveling novel opportunities for targeted therapy of GBC diagnosed during the implementation of prevention programs;
- exploiting existing and newly generated epidemiological and multi-omics data to improve early detection and optimize GBC prevention;
- contributing significantly to training the next generation of Latin American researchers in personalized GBC prevention.

## Data collected

### Study populations

**Figure 1.**
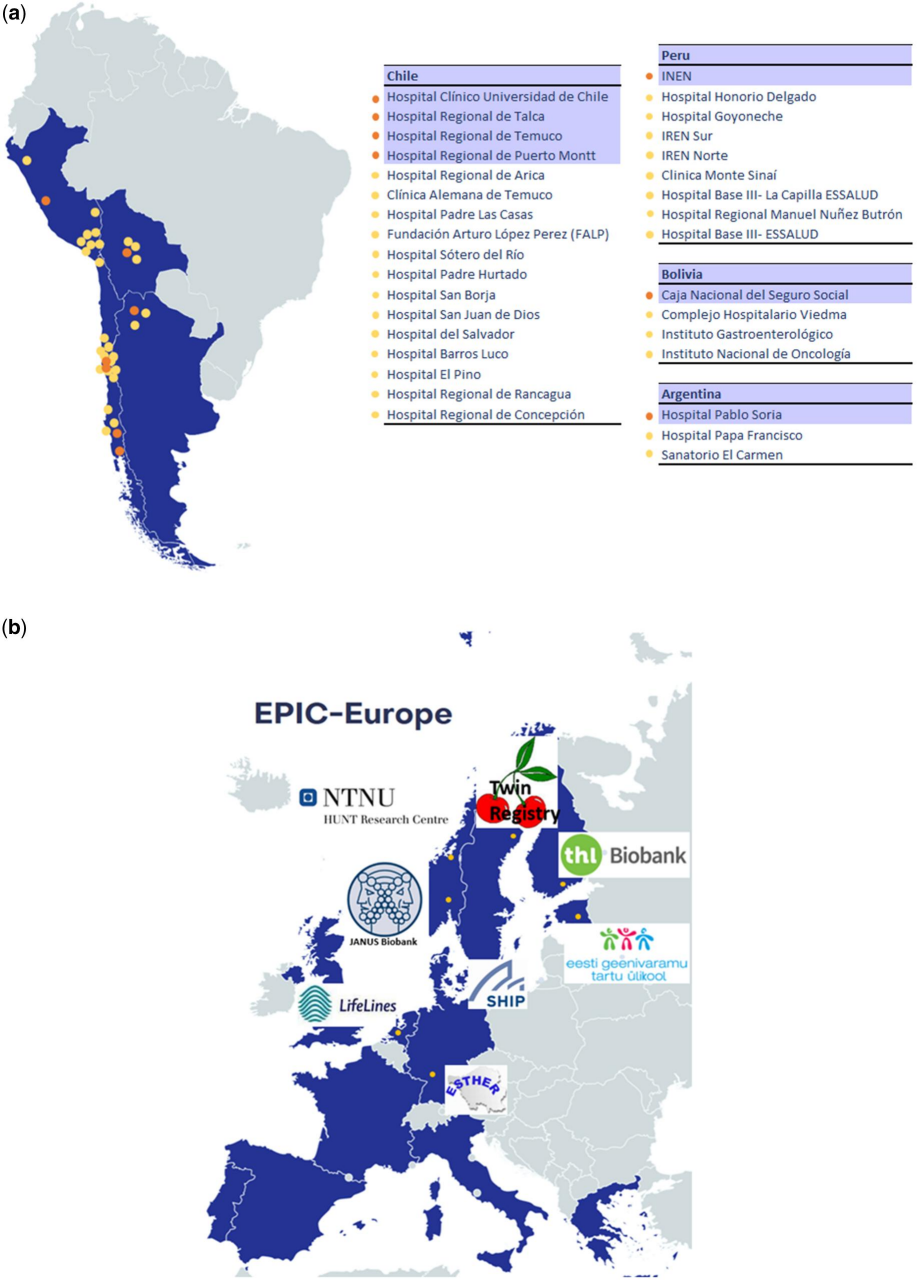
Participating countries in the EULAT Eradicate GBC. Patients affected by gallbladder cancer and gallstone disease are recruited in (a) four South American countries (Argentina, Bolivia, Chile, and Peru) and (b) Europe within the large European cohorts. Dark bullets: initial recruitment sites; light bullets: contingency recruitment sites. Maps created with MapChart.

The EULAT Eradicate GBC combines a multinational study of Latin American GBC cases and gallstone disease controls with multinational European cohorts, covering data and samples from patients in regions with high (Latin America) and low (Western Europe) GBC incidence (Fig. 1).

GBC cases comprise patients with a confirmed diagnosis of GBC (ICD-10 diagnosis code C23). The study includes only individuals aged >18 years and excludes patients with a porcelain gallbladder, polyps, non-cholesterol stones, or abnormalities of the pancreas or bile ducts. Gallstone disease controls without preneoplastic (gallbladder dysplasia) or neoplastic gallbladder lesions from the case–control study and controls from the cohort study are matched with the cases by sex, age, and BMI. The study protocol conforms to the ethical guidelines of the 1975 Declaration of Helsinki. All participants provide written informed consent before enrolment.

#### Patients from high-incidence regions in South America

**Figure 2.**
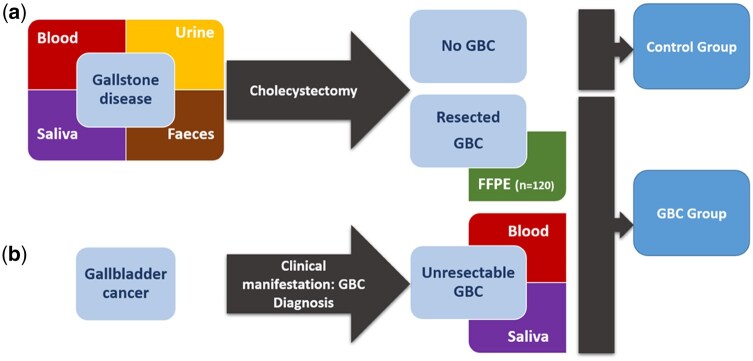
Recruitment strategy and samples collected in Latin America. Two types of patients are recruited: (a) presurgical samples and interview data are collected from patients with gallstones before cholecystectomy. After pathological review, we include patients with neoplastic findings or cancer in the case group. (b) Post-diagnostic samples and interview data are also collected from patients already diagnosed with GBC. Controls are selected from the group of cholecystectomized patients without (pre-)neoplastic gallbladder lesions and matched to GBC cases by age, sex, BMI, and recruitment center.

Two distinct types of GBC patients are recruited in Argentina, Bolivia, Chile, and Peru (Fig. 2). (i) Pre-surgical samples and interview data are collected from gallstone disease patients before cholecystectomy. Upon pathological review, 1%–3% of resected gallbladder specimens show preneoplastic or neoplastic lesions and are included in the GBC case population. (2) Post-diagnostic samples and data are collected from patients still carrying the primary GBC tumor (mainly patients with unresectable GBC from oncology and palliative care units).

Controls without preneoplastic or neoplastic lesions in the gallbladder are selected from the group of cholecystectomized patients and matched for sex, age, and BMI to the locally recruited GBC patients. In addition to sporadic GBC patients, we invite families with more than one member affected by GBC to participate in the study.

##### Patient recruitment process

Standard operating procedures (SOPs) are in place at each recruitment center to identify patients (i) undergoing cholecystectomy or (ii) diagnosed with GBC. Upon invitation by the physician, a trained study nurse provides eligible participants with detailed information about the study and answers any questions that the patient may have. If the patient agrees to participate and signs the informed-consent form, then the study nurse interviews the patient and collects blood, saliva, urine, and stool samples according to the EULAT Eradicate GBC SOPs and all ethical and legal regulations for biomedical research. The study nurse also invites relatives of GBC patients who report a family history of GBC during the interview [e.g. mother or sister(s) also affected by GBC] to participate in the study—after pathological confirmation of the diagnosis in the relative.

##### Electronic data collection with the application EULAT eCollect

We conduct patient interviews by using an application developed specifically for the EULAT Eradicate GBC project—EULAT eCollect [5]. EULAT eCollect ensures standardized data and sample collection across all recruitment centers and greatly facilitates monitoring local recruitment rates, and data and sample quality control. We organized EULAT eCollect into four separate modules (Table 1): Module 1—socio-demographic interview, Module 2—sample information, Module 3—case report form, and Module 4—food-frequency questionnaire [6].

**Table 1.** Module of the web application EULAT eCollect.

*Module*	*Collected data/information*
Module 1—Socio-demographic interview	This module permits the capture of information on established and potential risk factors for GBC. It includes study nurses measuring waist and hip circumference according to the guidelines of the Chilean National Health Surveys and administering structured questionnaires on demographic characteristics, tobacco and alcohol use, exposure to chemical pollutants, medical history (including gallstones, cholecystitis, inflammatory conditions, liver disease, and other gastrointestinal conditions), medication use, family history of cancer and gallstones, height and weight history, body fat distribution, diet, reproductive history, and lifestyle factors
Module 2—Sample information	This module facilitates the collection of relevant information on different types of specimens (blood, urine, feces, and saliva). The information collected includes transport, processing, and storage conditions, which significantly facilitates the automatic generation of preanalytical sample quality indicators according to biobank standards
Module 3—Case report form	This module is used to collect clinical and patient information, the characteristics of resected gallbladder specimens, and information about the patient's treatment. The study nurses coordinate clinical data abstraction with the participating hospitals. Information collected for the gallstone control group includes e.g. comorbidities, the dimensions of the resected gallbladder, and its contents (gallstones or sludge; number, size, and composition of gallstones). The information collected for GBC patients includes the type of GBC diagnosis, tumor stage at diagnosis and histological type, comorbidities, lymph node involvement, presence and location of any metastases, date, and type of surgery and treatment information
Module 4—Food-frequency questionnaire	This module permits the application of a food-frequency questionnaire, validated for Argentina, Chile, and Uruguay, to a subset of recruited patients [6]

We have designed and are constantly updating an IT platform to store the extensive socio-demographic, biospecimen, clinical, dietary, and molecular-genetic data generated within the EULAT Eradicate GBC to facilitate the monitoring of recruitment rates, quality of collected data, and preanalytical quality of collected samples by using Standard PREanalytical Codes (SPRECs) and to provide researchers with information on data and sample availability [7]. The IT platform is designed to fulfill the regulations, concepts, and technical recommendations that apply to the security and protection of personal data on the basis of the EU General Data Protection Regulation (GDPR), as well as the specific regulations in the Latin American beneficiary countries of the project (Argentina, Bolivia, Chile, and Peru). In addition to providing secure access to comprehensive anonymized biospecimen, epidemiological, and multi-omics information, we expect the IT platform to be a fundamental tool to strengthen collaboration and networking among all consortium members.

##### Biospecimen collection

We have established a continuously expanding biobank with samples from Latin American and European patients with gallstone disease and GBC. Following detailed SOPs, samples are collected and processed in >17 Chilean, 9 Peruvian, 4 Bolivian, and 3 Argentinean centers (sample specifications are presented in Table 2). Samples are aliquoted into 2D barcoded cryovials to ensure unambiguous sample identification and localization and stored at –80°C. One-third of the samples (series B) are stored locally as part of the EULAT Eradicate GBC biorepository and two-thirds (Series A) are sent to Heidelberg University for molecular-genetic characterization and long-term storage.

**Table 2.** Biospecimen collected within the EULAT Eradicate GBC.

*Sample Type*	*Collection*	*Volume Series A*	*Volume Series B*
Blood	Blood collection is coordinated within the clinical routine. After refrigerated transport to the laboratory and processing, serum, plasma, buffy coat, and whole-blood samples are aliquoted. Storage at –80°C	Whole blood: 2×1000 µl Serum: 3×1000 µl Plasma: 3×1000 µl Buffy coat: 1×1000 µl	Whole blood: 1×2000 µl Serum: 1×2000 µl Plasma: 1×2000 µl
Saliva	Saliva is collected by using the Oragene™ DNA Self-Collection Kit with storage at room temperature	4000 µl	NA
Urine	A midstream urine sample is collected from patients in a cup and stored at 4 °C until processing. Storage at –80°C	3×2000 µl	3×2000 µl
Feces	Trained EULAT Eradicate GBC study nurses coordinate the collection of fecal samples before cholecystectomy. Storage at –80°C	2×500 mg	2×500 mg
Tissue	Formalin-fixed paraffin-embedded tissue samples from a subgroup of patients are requested from the relevant pathology departments	NA	NA
Bile	Bile is collected from a subgroup of patients, aliquoted, and stored at –80°C	3×2000 µl	3×2000 µl

##### Recruitment centers and recruitment harmonization

Patient recruitment and sample processing are harmonized across all recruitment sites according to comprehensive SOPs and the EULAT eCollect application also facilitates standardized data and sample collection. Harmonization activities include training recruitment staff in biobanking and biorepositories, applying technical and operational guidelines, and establishing a program for quality assurance, quality control, and standardized data management.

The technical, operational guidelines in place include (i) guidelines on international biobanking best practices according to international organizations (OECD, 2009; ISBER, 2018; NCI, 2016), (ii) comprehensive SOPs describing all processes from study invitation and informed consent to collection, handling, processing, long-term storage, and shipment of biological samples, and (iii) guides on ethical, legal, and social issues (ELSI) [8].

The EULAT Eradicate GBC program on quality assurance, quality control, and standardized data management comprises sharing quality-control documentation with all recruitment sites and training recruitment staff to implement the quality-assurance/quality-control procedures partly reflected in the SPREC indicators.

Audits to ensure compliance with SOPs are performed at each recruitment site at the start, during patient recruitment, and after each sample shipment. In addition, a Latin American project manager regularly monitors all processes and operations by using automated reports.

#### Pre-diagnostic samples in patients from low-incidence regions of Europe

In addition, we are in the process of collecting samples and data from >200 prospective GBC case–control pairs nested in the large European prospective cohorts, including the Estonian Genome Project, the European Prospective Investigation into Cancer and Nutrition (EPIC), the Finnish FINRISK and Health2000 cohorts, the Norwegian Janus Serum Bank and the Helseundersøkelsen i Nord-Trøndelag (HUNT) study, the Dutch LifeLines cohort study, the Swedish Twin Registry and the German Epidemiological Study on Prevention, Early Detection and Optimised Therapy of Chronic Diseases in the Elderly Population (ESTHER), the Cooperative Health Research in the Region of Augsburg (KORA), the Study of Health in Pomerania (SHIP), and the Heinz Nixdorf Recall studies [9–19]. This collaborative effort has resulted in a unique collection of prospective DNA, serum, and plasma samples, and extensive, high-quality lifestyle, environmental exposure, and molecular-genetic data, which we hope to expand with contributions from other studies.

## Data resource use

From January 2020 (first patient in) to March 2025, 11 453 Latin American participants were enrolled in the study (Table 3).

**Table 3.** Characteristics of the Latin American case–control study population.

Variable	Category	*n*	Percent
Age (years): median (range)	48 (18–96)		
Sex	Female	8497	74
	Male	2956	26
Country	Argentina	274	2
	Bolivia	1570	14
	Chile	7829	68
	Peru	1780	16
BMI (kg/m^2^)	<25	2602	23
	25–29.9	4227	37
	30–34.9	2717	24
	≥35	1619	14
	Missing	288	3
Gallbladder disease	Gallbladder cancer	1033	9
	Dysplasia	391	3
	Gallstone disease	10 029	88
Family history of GBC	Yes	288	3
	No	10 099	88
	No information	1066	9
Self-reported ancestry	Quechua	1489	13
	Mapuche	1297	11
	Aymara	554	5
	Other	8016	70
	No information	97	1
Health insurance	Public	7598	66
	Private	1664	15
	Other	2148	19
	No information	43	0
Education	No formal schooling	229	2
	Low education	2140	19
	Medium education	4261	37
	High education	4692	41
	No information	131	1
Alcohol consumption	Never	109	1
	Once a month or less	3608	32
	2–4 times a month	1328	12
	2–3 times per week	191	2
	≥4 times per week	104	1
	No information	6113	53
Smoking	Yes	1276	11
	Occasionally	680	6
	No, but previously	2398	21
	No	7010	61
	No information	89	1
Fish/seafood intake	More than once per week	1271	11
	Once per week	4433	39
	<3 times per month	2472	22
	Less than once per month	3158	28
	No information	119	1
Diabetes	Yes	1433	13
	No	9768	85
	No information	252	3
Occupational exposure to pesticides	Yes	673	6
	No	10 422	91
	No information	358	4

Most individuals were recruited in Chile (68%), followed by Peru (16%), Bolivia (14%), and Argentina (2%). The median age of the study population is 48 years and 74% of the participants are female. In terms of Indigenous ancestry, Quechua is the most represented (13%), followed by Mapuche (11%) and Aymara (5%). The majority of individuals recruited (77%) are overweight (BMI ≥ 25 kg/m^2^) and 38% are obese (BMI ≥ 30 kg/m^2^). GBC or gallbladder dysplasia was diagnosed in 12% of the study population.

In addition, >400 participants from the European prospective cohorts have been included in the study.

Using these datasets, we have conducted several studies on GBC risk prediction, biomarker identification, and causal mechanisms underlying GBC development.

For example, we have shown the potential of individual genotype data to predict long non-coding (lnc)RNA expression in serum through lncRNA preselection, *cis*-expression quantitative trait loci (eQTL) validation, and association analysis between genotype-based lncRNA expression and GBC risk [20].

In separate analyses of the DNA methylation of tissue samples from patients affected by gallstones, gallbladder dysplasia, and GBC, we observed increased epigenetic alterations and copy number variations with sequential disease progression from gallstones to inflammation, dysplasia, and GBC, mainly involving the hypermethylation of cytosine–guanine dinucleotide islands and gene promoter regions [21].

In another study, through Mendelian randomization, we demonstrated a direct causal genetic link between BMI and GBC risk in the Chilean population and, instead, in the European population, an indirect effect mediated by gallstones [2]. In another study, we observed an unconfounded association between the individual proportion of Indigenous American Mapuche ancestry and GBC risk, which was also mediated by gallstones [22].

We designed and validated a 25-gene sequencing panel, which permitted the identification of several GBC predictive biomarkers (*PARP*, *EGFR*, *PIK3CA*, *mTOR*, and Hedgehog signaling inhibitors). Targeted gene panels may represent a cost-effective alternative for implementing real-world precision medicine in Latin America [23].

In a retrospective study of 473 Chilean GBC patients and 2137 population-based controls, we developed and internally validated three prediction models for GBC risk. The baseline model, which only included gallstones, sex, and birth year, performed worst with an area under the precision-recall curve of 0.44% (95% confidence interval (CI) 0.42–0.46), which increased by 0.22 (95% CI 0.15–0.29) with the inclusion of nongenetic factors (Enhanced Model I: BMI, education, Mapuche surnames, number of children, family history of GBC). The additional incorporation of genetic factors in the Enhanced Model II further improved the risk prediction by 2%. The consideration of additional information permitted the number of cholecystectomies needed to prevent one GBC case to be reduced from 115 in the baseline model to 80 in Enhanced Model II [24].

A up-to-date list of EULAT Eradicate GBC publications can be found at https://www.klinikum.uni-heidelberg.de/eulat-eradicate-gbc/publications.

## Strengths and weaknesses

For various reasons, including sociopolitical and geographical ones, GBC remains a neglected disease due to a lack of research resources and efforts. Despite the aggressive nature of the disease, the treatment options remain very limited. We believe that accurate risk prediction and early detection can reduce GBC mortality in low-income, high-incidence regions. To achieve this goal, we have established the EULAT Eradicate GBC, funded by the Horizon 2020 EU research and innovation program (https://cordis.europa.eu/project/id/825741).

The main strengths of this resource are the representativeness of the patients recruited in Argentina, Bolivia, Chile, and Peru [of the 10 434 cases of GBC diagnosed in Latin America and the Caribbean in 2022 according to Globocan, 4833 (46%) were diagnosed in the four participating countries], the large study size (as of May 2024, the number of patients recruited was 11 453), and the sample collection prior to cholecystectomy in gallstone disease patients (most biospecimens are treatment-naive). Furthermore, the interviews remain uninfluenced by a possible cancer diagnosis, thus most likely reflecting the patients’ lifestyles. The sample and data collection within the EULAT Eradicate GBC include blood, urine, feces, bile, and saliva.

Standardized operating procedures ensure harmonized methods of data and sample collection at all recruitment sites and the comparability of the generated data. This harmonization also includes the central organization of laboratory materials, such as barcoded cryotubes, which guarantee sophisticated biobanking of the study samples.

Furthermore, the multinational study design combining a case–control study with a prospective cohort enables researchers to discover disease biomarkers in the case–control design, which can then be validated in the prospective cohort. This, however, relies on the assumption that biomarker profiles are similar across study designs and are not masked by gallstone disease in the case–control group.

One limitation of the study is the independently developed SOPs for data and sample collection in the European cohorts, which require careful harmonization before data analysis. Finally, a large number of gallstone disease patients need to be enrolled to recruit each case of GBC: among all cholecystectomized patients, only 1%–2% are diagnosed with GBC on histopathological examination. Currently, 115 cholecystectomies need to be performed to prevent each case of GBC, demonstrating the urgent need to develop personalized strategies to identify high-risk individuals.

## Data resource access

By establishing the EULAT Eradicate GBC, we aim to address the challenges of GBC prevention and advance the eradication of preventable GBC through the integration of a European–Latin American GBC biorepository into an IT platform with an unsurpassed collection of data and samples.

The EULAT Eradicate GBC is governed by an executive board and managed by a project management office. Data and samples are available for research purposes only. Research collaboration requests and research proposals for access to data and samples are welcome and can be addressed to EULAT_Eradicate_GBC@imbi.uni-heidelberg.de. Requests for access to data and samples are assessed on the basis of scientific quality, public benefit, medical ethical considerations, compliance with the GDPR, and the risk of identification of the study participants. A processing fee may be charged for the delivery of samples and data.

## Ethics approval

This study was performed in line with the principles of the Declaration of Helsinki and has been approved by the ethics committees of Servicio de Salud Metropolitano Oriente, Servicio de Salud Metropolitano Sur Oriente, and Servicio de Salud Metropolitano Central in Santiago de Chile; Servicio de Salud Coquimbo in Coquimbo, Chile; Servicio de Salud Maule in Talca, Chile; Universidad Católica del Maule in Talca, Chile; Servicio de Salud Concepción in Concepción, Chile; Servicio de Salud Araucanía Sur in Temuco, Chile; Servicio de Salud Valdivia in Chile, Centro de Bioética Universidad del Desarrollo and Clínica Alemana de Santiago in Santiago de Chile; Unidad de Investigación Hospital San Juan de Dios in Santiago de Chile; the Medical Faculties of Universidad de Chile and Pontificia Universidad Católica de Chile, Facultad de Medicina, Universidad Mayor de San Simón, Bolivia, Comité Provincial de Ética de Investigación de la Provincia de Jujuy, Argentina; and Comité de ética e Investigación del Instituto Nacional de Enfermedades Neoplásicas, Peru.
